# Measurement of the Length of Installed Rock Bolt Based on Stress Wave Reflection by Using a Giant Magnetostrictive (GMS) Actuator and a PZT Sensor

**DOI:** 10.3390/s17030444

**Published:** 2017-02-23

**Authors:** Mingzhang Luo, Weijie Li, Bo Wang, Qingqing Fu, Gangbing Song

**Affiliations:** 1 Electronics and Information School, Yangtze University, Jingzhou 434023, China; luomingzh@163.com (M.L.); jpufqq@yangtzeu.edu.cn (Q.F.); 2 Department of Mechanical Engineering, University of Houston, Houston, TX 77004, USA; wli27@uh.edu (W.L.); gsong@uh.edu (G.S.); 3 School of Civil Engineering, Dalian University of Technology, Dalian 116023, China; 4 Key Laboratory of Transportation Tunnel Engineering, Ministry of Education, Southwest Jiaotong University, Chengdu 610031, China

**Keywords:** rock bolt monitoring, determination of installed rock bolt length, giant magnetostrictive actuator, stress wave reflection, instant phases, non-destructive evaluation

## Abstract

Rock bolts, as a type of reinforcing element, are widely adopted in underground excavations and civil engineering structures. Given the importance of rock bolts, the research outlined in this paper attempts to develop a portable non-destructive evaluation method for assessing the length of installed rock bolts for inspection purposes. Traditionally, piezoelectric elements or hammer impacts were used to perform non-destructive evaluation of rock bolts. However, such methods suffered from many major issues, such as the weak energy generated and the requirement for permanent installation for piezoelectric elements, and the inconsistency of wave generation for hammer impact. In this paper, we proposed a portable device for the non-destructive evaluation of rock bolt conditions based on a giant magnetostrictive (GMS) actuator. The GMS actuator generates enough energy to ensure multiple reflections of the stress waves along the rock bolt and a lead zirconate titantate (PZT) sensor is used to detect the reflected waves. A new integrated procedure that involves correlation analysis, wavelet denoising, and Hilbert transform was proposed to process the multiple reflection signals to determine the length of an installed rock bolt. The experimental results from a lab test and field tests showed that, by analyzing the instant phase of the periodic reflections of the stress wave generated by the GMS transducer, the length of an embedded rock bolt can be accurately determined.

## 1. Introduction

Rock bolts are widely used as the primary reinforcing members in stabilizing engineering structures, such as underground excavations, slopes, retaining walls, tunnels, and dam foundations. Rock bolts are steel studs that are bolted into the rock mass to prevent the movement and expansion of rock strata and, thus, improving the stability of the structure. Given the importance of rock bolts, it is crucial to evaluate the properties and conditions of installed rock bolts. The independent inspection of the lengths of installed rock bolts is very important to ensure the installed rock bolts meet the design requirements. If the installed rock bolt length does not meet the design requirements, failures of the reinforced structure could happen and lead to economic and environmental catastrophic losses. It is, therefore, necessary to measure the length of installed rock bolts in order to ensure the safe operation of the reinforced structure. Due to the embedded nature of the most part of a rock bolt, determination of the length of an installed rock bolt is a challenge. 

The study of using an ultrasonic guided wave method for non-destructive evaluation of rock bolts has been researched [1,2,3,4,5,6,7,8,9,10,11]. In recent years, the stress wave reflection method has emerged as a promising non-destructive evaluation technique for rock bolts condition assessment [12,13,14]. The stress wave reflection method, which is a type of quick and effective non-destructive evaluation method for rock bolt condition assessment, relies on the basis of the one-dimensional wave propagation theory. Normally, the stress wave reflection method involves wave frequencies that are lower than that of ultrasound. In theory, a rock bolt can be considered as a one-dimensional linear elastic rod. When the free end of the bolt is excited to generate a stress wave, the stress wave will propagate along the rock bolt. If there is any discontinuous interface between the bolt and the rock mass, such as the embedded bottom end of the bolt, necking, deformation, or reduced cross-section due to corrosion, and missing grout, there will be a change in the stress wave parameters of the reflected wave, such as amplitude, phase, frequency, and arrival time. Based on the parameters of the reflected wave, it is able to determine the length and defects of a rock bolt.

However, the stress wave reflection method suffers from erroneous assessment issues in practical engineering. Firstly, the signal quality of the waveforms acquired from in situ testing is generally poor. The transducer used for stress wave generation has great influence on the quality of the acquired signals. To date, lead zirconate titanate (PZT) elements and hammer impacts are the traditional methods to excite stress wave in a rock bolt [6,10]. These methods show their limitations when it comes to practical application. The hammer impact method requires highly trained personnel to ensure the consistency of stress waves for each impact in terms of energy, frequency, and phase. The piezoelectric element cannot generate enough energy to guarantee multiple periodic reflections of the stress wave before the waves are fully attenuated. Secondly, experience-based interpretation of the rock bolt conditions is deeply influenced by the experience of the personnel. In order to solve this problem, Starkey et al. [15] developed a rock bolt anchorage post-tension level diagnosis method based on neural networks. The lumped parameter dynamic model was used to describe the relationship between the general frequency and the tension level of the rock bolt, and was then used to produce a training dataset for the neural network. The trained neural network was used to diagnose the rock bolt condition from experimental data. However, the neural network required a large computational load and processing capability, which was not suitable for in situ real-time diagnosis. 

In recent years, the giant magnetostrictive (GMS) materials are widely used in many fields and tend to replace the piezoelectric materials due to their advantages of large deformation ratio, high energy conversion rate, rapid response, good reliability, wide bandwidth, and ease to actuate [16,17,18,19,20,21,22,23,24,25,26]. The interpretation of the data obtained from the GMS transducer plays important role in assessing the condition of the tested structures accurately. Zhang et al. [27] applied the wavelet transform analysis to extract the rock bolt length information. The stress wave was decomposed into components of different frequencies. They found that the damage information of the rock bolt were concentrated in one or several frequency bands, which can be used to improve the damage assessment accuracy. Luo et al. [28] examined the efficacy of determining the rock bolt defects and length through finding the discontinuity points in the instant phase of the stress wave. 

In this paper, a new type of stress wave generation method was proposed based on the GMS transducer. The GMS transducer was able to generate enough energy for the stress wave to be reflected along the rock bolt multiple times. The reflected wave was then detected by a PZT sensor. The sensor was connected to a data acquisition device, which was capable of recording the overall stress wave signals of multiple periodic reflections. A new integrated signal processing method, including correlation analysis, wavelet denoising, and Hilbert transform, was developed to analyze the reflected stress waves. This new method is simple but effective. The effectiveness of the proposed method was validated through both lab studies and field measurements, which showed that the proposed method was able to measure the rock bolt length accurately. 

## 2. Proposed Instrumentation and Method

### 2.1. Measurement System Setup

**Figure 1:**
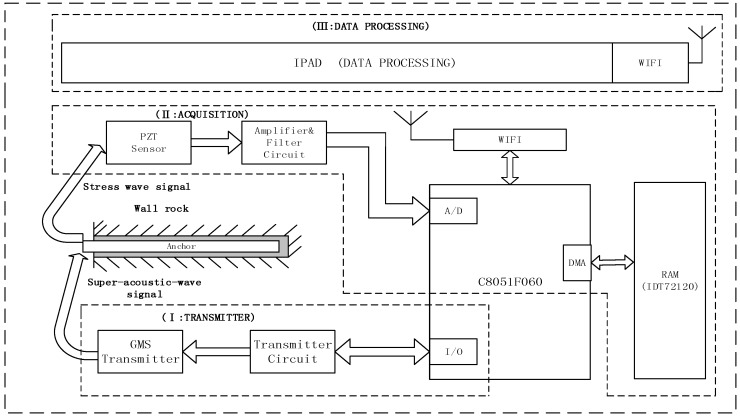
Measurement system setup.

Figure 1 illustrates the setup of the measurement system. A giant magnetostrictive (GMS) transducer and a PZT sensor were attached to the exposed end of the rock bolt. The GMS transducer was used to generate stress wave along the rock bolt and the PZT sensor was used to receive the reflected multiple periodic stress wave. As shown in Figure 1, the microcontroller (C8051F060, Silicon Laboratories Inc, Austin, TX, USA) is the key component of the measurement system, and has three major functions. First, the microcontroller controls the transmitter circuit that actuates the GMS transmitter. Three types of output signals: the single pulse output, the pulsed coding output, and the sweep sine wave output, can be generated. Second, the signals from the PZT sensor were acquired by the A/D module of the microcontroller, and then stored into RAM (IDT71V124SA, Integrated Device Technology, San Jose, CA, USA) via direct memory access (DMA). Third, the data were transferred to a tablet via a Wi-Fi module and processed in real-time.

### 2.2. Principle of GMS Transducer

**Figure 2:**
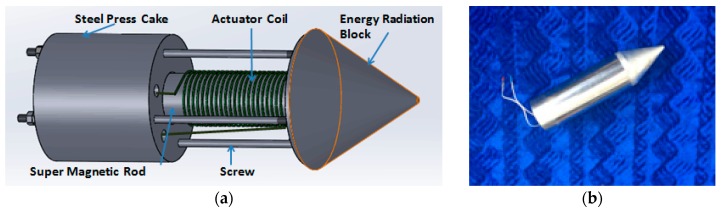
Illustration and photograph of the GMS transducer: (**a**) components of the GMS transducer; and (**b**) photo of the GMS transducer.

The developed giant magnetostrictive transducer consists of a super magnetic rod, an actuator coil, a steel press cake, screws, and an energy radiation block. Figure 2a shows the component of the GMS transducer and Figure 2b shows a photo of the GMS transducer. The super magnetic rod acted as an oscillating element. The super magnetic rod was covered with protective coating before being placed in the center of the actuator coil. If an alternating current is applied to the actuator coil, the super magnetic rod will oscillate along its axial direction. The oscillation will be transferred to the object under test via the cone shaped energy radiation block. The energy and frequency of the generated stress wave can be adjusted by changing the parameter of the input alternating current. The fabricated GMS transducer is weighted 1 kg, whose instant voltage can range from 12 V to 600 V and the excitation frequency can range from 100 Hz to 15,000 Hz. The maximum power output can reach 2000 W. 

**Figure 3:**
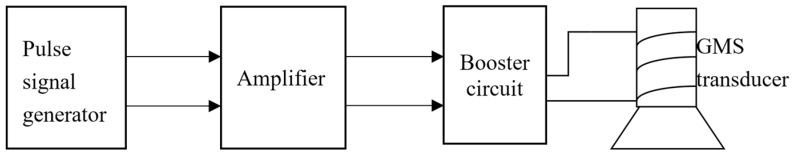
The pulse driving circuit for the GMS transducer.

Figure 3 shows the pulse driving circuit for the GMS transducer. The pulse signal was generated by the microcontroller, then amplified by the amplifier. The amplified pulse signal was boosted by the booster circuit. For example, a 12 V signal can be boosted to 150 V, and 300 VPP for a bipolar pulse. The boosted pulse was fed into the GMS transducer to generate a stress wave. The pulse generated includes unipolar and bipolar pulses. The selection of the polarity of the pulse depends on the experience. 

### 2.3. Algorithm for Rock Bolt Length Information Extraction

In this paper, we develop a new integrated algorithm for the rock bolt length extraction from the reflected stress wave signals, and the algorithm can be divided into three steps, which include the correlation analysis, wavelet denoising, and Hilbert transform. The algorithm is simple and effective, and can be easily implemented on the portable device, as shown in Figure 1.

***Step 1****: Perform correlation analysis to select the signals with good consistency.*

Several repeated measurements will be performed for the same rock bolt. The acquired reflected stress wave signals are represented by $S_{i}\left(n\right)$, i=1,2,……N. $S_{i}\left(n\right)$ is the ith measurement for the same rock bolt. If the output circuit and the data acquisition module are stable, the peak and the location the peak of the correlation $r_{S_{i}S_{j}}(m)$ between any two of the measurements will lie in a small range. If we define:

(1) $$r_{S_{i}S_{j}}(m)=\overset{+\infty }{\underset{-\infty }{\sum }}S_{i}\left(n\right)S_{j}\left(n-m\right)$$

where $r_{S_{i}S_{j}}(m)$ is the correlation between any two different measurements for the same rock bolt. Analyzing the peak value of $r_{S_{i}S_{j}}(m)$, it is possible to select the measurements with the best correlation, which eliminates the measurement errors induced by human or the environment. For the selected measurements, they can be superimposed into one waveform S(n) by slightly adjusting their waveform position to ensure that they are well-aligned. The processed signal will have a better signal-to-noise ratio.

***Step 2:**** Denoise the signal using wavelet analysis.*

The Mallat wavelet denoising algorithm is used to decompose, reconstruct and finally remove the noise of the signal. The Mallet algorithm [29] decomposes a signal f(t) into a series of sub-bands. The frequency ranges of the sub-bands are related to the sampling frequency of the signal. Suppose the sampling frequency of the original signal is $f_{s}$ and the decomposition scale is J, the dominant frequency of the stress wave after Fourier transform is $f_{m}$, and the frequency range of useful signal is $\left[\frac{f_{m}}{N},\;Nf_{m}\right],N\in \left(2,3,4,5,6,7,8,9\right)$. In order to remove low frequency interference, the decomposition scale J needs to satisfy the following expression:

(2) $$\frac{1}{2^{J+1}}f_{s}\leq \frac{f_{m}}{N}$$

Rearranging the above expression, J can be expressed as follows:

(3) $$J\geq \log_{2}\frac{Nf_{s}}{f_{m}}-1$$

For the frequency bands outside the range of $\left[\frac{f_{m}}{N},\;Nf_{m}\right]$, the signal will be set to zero when reconstructing the denoised signal. For the frequency bands that lie within the range of $\left[\frac{f_{m}}{N},\;Nf_{m}\right]$, the wavelet coefficients will be quantified using the unbiased risk estimate principle to select the proper thresholds. Finally, the Mallat algorithm is used to reconstruct the denoised signal.

***Step 3:**** Obtain instant phases of the reflected waves using Hilbert transform.*

An analysis of the periodic discontinuities in the instant phases provides the information about the length of the rock bolt.

The signal of the reflected stress wave satisfies the following equation:

(4) $$S\left(t\right)=\{\begin{array}{c} x_{1}\left(t\right)\;0\leq t<T\\ x_{1}\left(t\right)+x_{2}\left(t\right)\;t\geq T \end{array}$$

where $x_{1}\left(t\right)$ is the excitation stress wave from the coupling of the transmitter and the rock bolt end, $x_{2}\left(t\right)$ is the reflected stress wave, and T is the arrival time of the first reflected wave. Assume the Hilbert transform of $x_{1}\left(t\right)$ and $x_{2}\left(t\right)$ are $y_{1}\left(t\right)$ and $y_{2}\left(t\right)$, respectively. From the relation $\theta \left(t\right)=\tanh^{-1}\frac{y\left(t\right)}{x\left(t\right)}$, the instant phase θ(t) of the stress wave signal should satisfy the following equations [30]:

(5) $$\underset{t\to T^{-}}{\lim}\theta \left(t\right)=\;\tanh^{-1}\frac{y_{1}\left(t\right)}{x_{1}\left(t\right)}$$

(6) $$\underset{t\to T^{+}}{\lim}\theta \left(t\right)=\;\tanh^{-1}\frac{y_{1}\left(t\right)+y_{2}\left(t\right)}{x_{1}\left(t\right)+x_{2}\left(t\right)}$$

(7) $$\underset{t\to T^{-}}{\lim}\theta \left(t\right)\neq \underset{t\to T^{+}}{\lim}\theta \left(t\right)$$

If a sudden change in the stress wave at the *N*-th sampling point due to bottom reflection or missing grout, the location of reflection, w, which could be the location of the bottom surface or the location of the missing grout, is given by:

(8) $$w=\frac{N\;\Delta t\;v}{2}$$

where Δt is the sampling interval of the data acquisition system, and v is the propagation velocity of the stress wave along the longitudinal direction of the rock bolt. The discontinuity points in the instant phase can either be the location of defects or the bottom end of the rock bolt. In order to differentiate whether a reflection comes from defects or bottom end, a careful attention should be paid the characteristics of the instant phase. 

If enough energy is generated from the transmitter, the phase of the received signal will present periodic sudden change due to the reflections from defects and bottom end of the rock bolt. If no defects are presented in the rock bolt, the reflection is solely caused by the bottom end reflection, and the length of the rock bolt is calculated by Equation (9). However, if defects are presented, such as necking, deformation, reduced cross-section due to corrosion, and missing grout, the bottom reflection amplitude will be attenuated and the instant phase will be altered. Examining the periodic pattern of the multiple reflections, the rock bolt length can be easily identified.

The period of the sudden change in phase of the signal is equal to the traveling time of the stress wave propagating along the rock bolt. Let the period of the sudden change in phase be ΔT, then the length of the rock bolt is given by:

(9) $$l=\frac{\Delta T\;v}{2}$$

## 3. Experimental Verification

### 3.1. Specimen Fabrication

**Figure 4:**
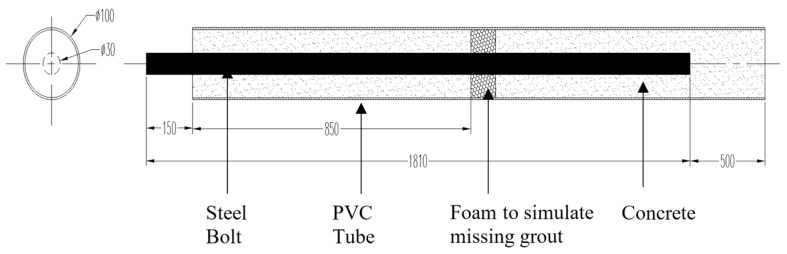
Diagram of the rock bolt specimen.

The rock bolt specimen was fabricated in laboratory. Figure 4 shows the schematic diagram and dimension details of the specimen. The rock bolt is a ribbed rebar which has a diameter of 30 mm and a length of 1810 mm. The rock bolt was placed in the center of a PVC tube. The PVC tube has a diameter of 100 mm and a length of 2150 mm. After the rock bolt was put in place, the PVC tube was filled with concrete. It is worth noting that a small segment of foam was placed within the PVC tube at a pre-determined location, as shown in Figure 4. The foam was used to simulate missing grout, which is a common defect during the rock bolt installation. In the later section, we will show that the proposed method can accurate estimate the length of the rock bolt even with the presence of a defect during the rock bolt installation.

### 3.2. Instrumentation and Experimental Setup

**Figure 5:**
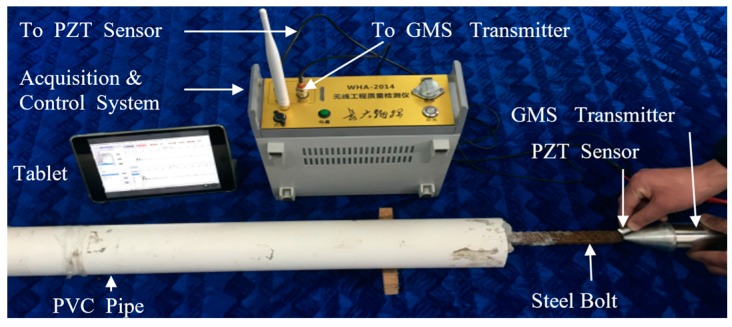
Instrumentation and experimental setup.

Based on the measurement scheme shown in Figure 1, all hardware, including the GMS transmitter (actuator), the PZT sensor, the data and control acquisition system, and the wireless display (a tablet), were set up, and Figure 5 shows the instrumentation and the experimental setup for rock bolt length estimation. The excitation signal for the GMS transducer was a unipolar pulse, which has an amplitude of 80 V and a duration of 100 μs. The fabricated GMS transducer has the best energy conversion rate when it is excited at the frequency of 10 kHz, which corresponds to a 100 μs duration pulse. Such characteristic is dependent on the size of the GMS transducer and the tightness of the screws. The PZT sensor was used to receive the reflected multiple periodic stress wave. The sampling frequency for data acquisition is 1 MHz. The measurement was repeated five times.

### 3.3. Data Analysis and Information Extraction

**Figure 6:**
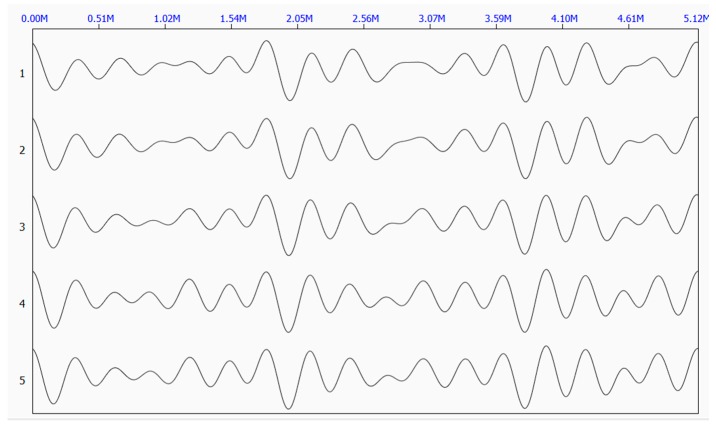
Original waveforms of the reflected stress wave.

**Figure 7:**
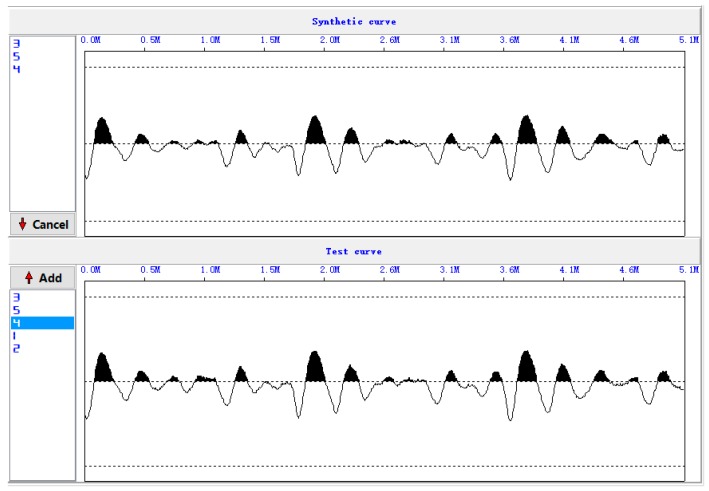
Superimposed waveforms.

Figure 6 shows the five original waveforms of the reflected stress wave. Figure 7 shows the superimposed waveform using the three most correlated waveforms (waveform 3, waveform 5, and waveform 4).

**Figure 8:**
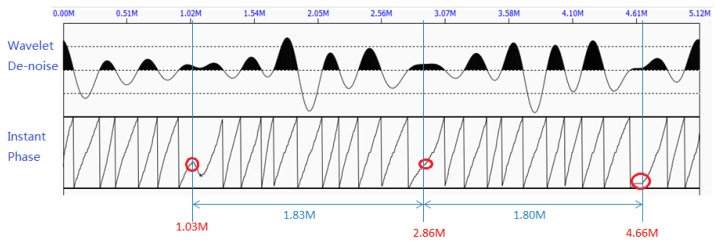
The denoised waveform by wavelet denoising algorithm (**top**) and the periodic pattern of the instant phase (**bottom**).

Figure 8 shows the denoised waveform after being processed by the wavelet denoising method, along with the periodic pattern of the instant phase. The locations of the sudden change of the instant phase can be calculated using Equation 8. The calculated locations are: $w_{1}=1.03\;m$, $w_{2}=2.86\;m$, $w_{3}=4.66\;m$, which are marked with red circles. This can be due to defects or bottom end reflection. Since $w_{2}-w_{1}\approx w_{3}-w_{2}$, we identified that such a change in the instant phase is due to the end reflections. Using Equation (9), the approximated length of the rock bolt is 1.815 m, which is close to the actual length of 1.810 m. This experiment in a lab setting clearly demonstrates the capacity of proposed method to determine the length of an embedded rock bolt, even in the presence of missing grout, which will also cause reflection. 

## 4. Field Tests

### 4.1. Site 1

**Figure 9:**
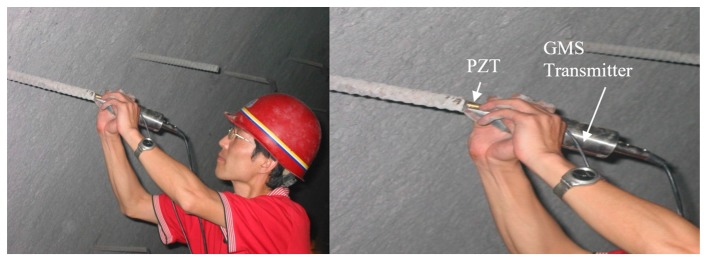
Field tests at Site 1.

**Figure 10:**
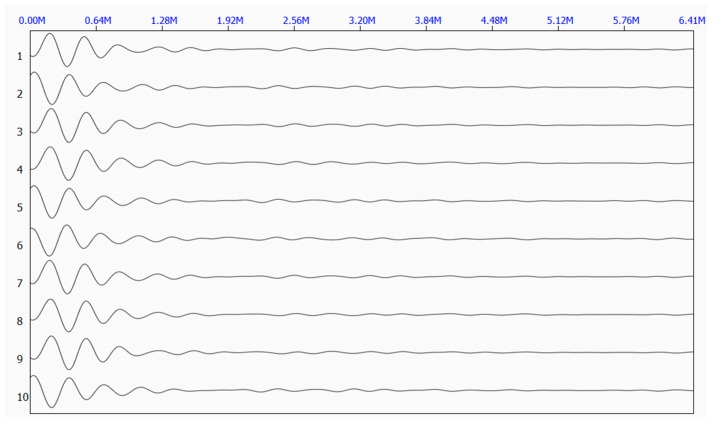
Original waveforms of the reflected stress wave at Site 1.

**Figure 11:**
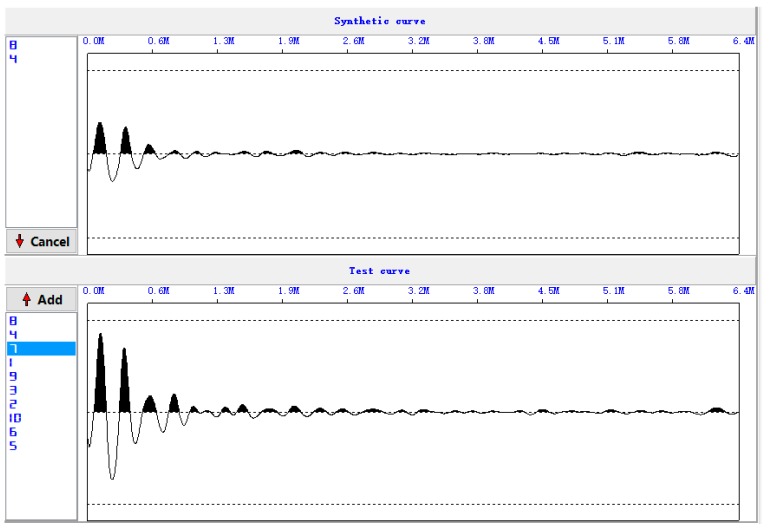
Superimposed waveforms at Site 1.

**Figure 12:**
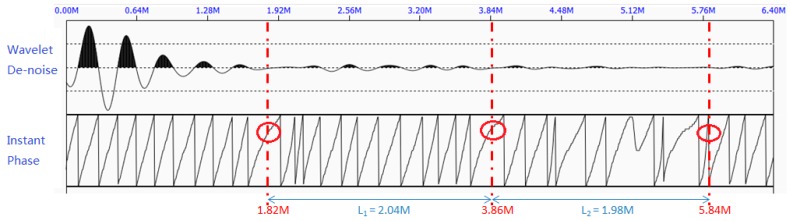
The denoised waveform by wavelet denoising algorithm (top) and the periodic pattern of the instant phase (bottom) at Site 1.

On 26 April 2016, a series of field tests were performed in a highway tunnel. The highway tunnel was located in Wuqi County, Yanan City, Shaanxi Province, China. The giant magnetostrictive (GMS) actuator has the best energy conversion rate at frequency around 10 kHz. This property is dependent on the sizes of the components of the GMS actuator. In practice, properly lowering the excitation frequency can reduce the attenuation of the stress wave. The selected excitation frequency should ensure the excited stress wave has multiple reflections. Therefore, the excitation signal was a unipolar pulse which has a dominant frequency at 7.8 kHz. The sampling frequency was 1 MHz. During the field tests, 50 rock bolts were randomly chosen. Measurement for each rock bolt was repeated 10 times. In addition, in situ pullout tests were performed for five of the chosen rock bolts to verify the measurement accuracy of the proposed rock bolt length measuring method. Table 1 lists the results of the five rock bolts, which shows that the proposed method is highly accurate in determining the length of installed rock bolts. Measurements from one of the rock bolts were shown. This rock bolt had a length of 2 m according to the pullout test. Figure 9, Figure 10, Figure 11 and Figure 12 show the steps and data analysis results. 

***Step 1:**** Data acquisition in the field.*

***Step 2****: Correlation analysis and superimpose.*

***Step 3****: Wavelet denoising and phase extraction.*

As can be seen from Figure 12, the sudden changes in the instant phase are periodically appeared in 1.82 m, 3.86 m, and 5.84 m. They are marked in red circle in the figure. Therefore, the measured length of this rock bolt is 2.01 m, which is very close to its actual length of 2 m. 

### 4.2. Site 2

**Figure 13:**
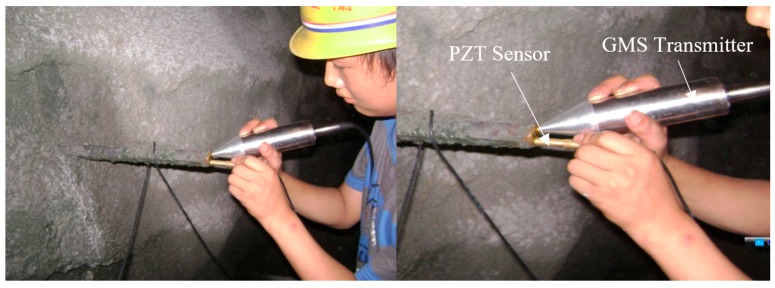
Field tests at Site 2.

**Figure 14:**
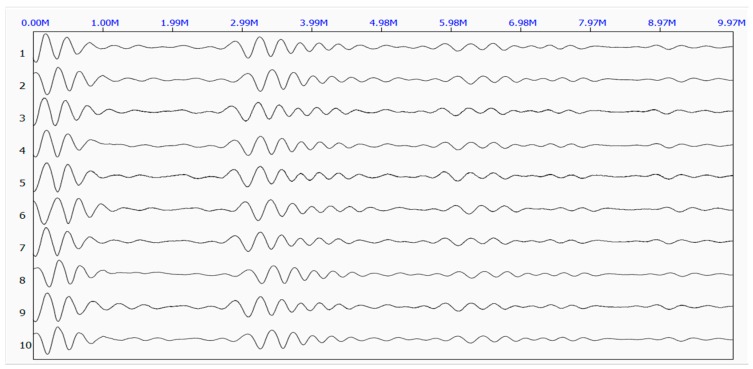
Original waveforms of the reflected stress wave at Site 2.

**Figure 15:**
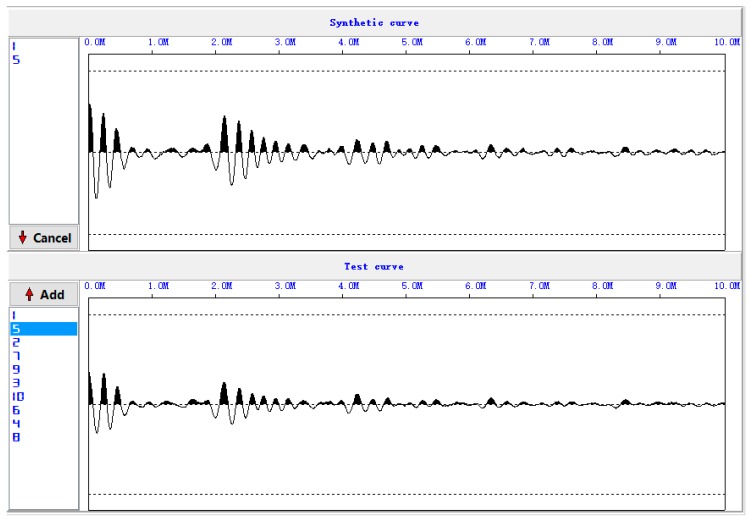
Superimposed waveforms at Site 2.

**Figure 16:**
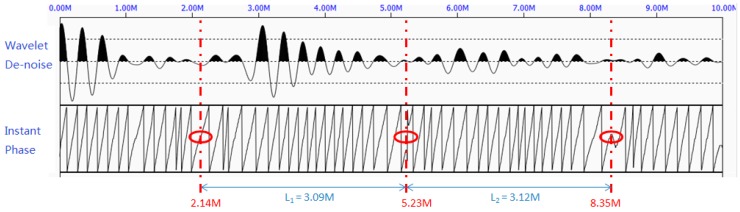
The denoised waveform by wavelet denoising algorithm (**top**) and the periodic pattern of the instant phase (**bottom**) at Site 2.

On 4 June 2016, another series of field tests were performed at the Yangqu hydropower station located in Xinghai County, Qinghai Province, China. The purpose of the tests was to assess the anchorage quality of the rock bolts. The adopted excitation source was a unipolar pulse at a dominant frequency of 8.8 kHz. The sampling frequency was set at 1 MHz. At this testing site, 30 rock bolts were randomly chosen. Measurement for each rock bolt was repeated 10 times. Additionally, in situ pullout tests were performed for five of the chosen rock bolts to verify the measurement accuracy of the proposed rock bolt length measuring method. Table 2 lists the results of the five rock bolts. Again, it shows that the proposed method is highly accurate in determining the length of installed rock bolts. The measurement steps for one of the rock bolt, which had an actual length of 3.1 m according to the pullout test, was shown. Figure 13, Figure 14, Figure 15 and Figure 16 show the steps and data analysis results. 

***Step 1****: Data acquisition in the field.*

***Step 2****: Correlation analysis and superimpose.*

***Step 3:**** Wavelet denoising and phase extraction.*

As can be seen from Figure 16, the sudden changes in the instant phase are periodically appeared in 2.14 m, 5.23 m, and 8.35 m. They are marked in red circles in the figure. Therefore, the measured length of this rock bolt is 3.15 m, which is very close to its actual length of 3.1 m.

## 5. Conclusions

This paper investigated the application of stress wave reflection method in assessing the length of rock bolts. The giant magnetostrictive transducer was used to excite stress wave along rock bolt in order to provide enough energy for the stress wave to be reflected multiple times. A PZT sensor was used to detect the reflected waves. A specialized measurement system was built. A new integrated three-step signal processing procedure was developed to extract the length information of the rock bolt. First, correlation analysis was performed on the waveforms to identify the most correlated waveforms and eliminate human and environment errors. Second, the superimposed waveform was denoised by a wavelet algorithm. Third, Hilbert transform was adopted to extract the useful information about rock bolt length and defects based on the periodic pattern of the phase change over the multiple reflections. The signal processing procedure was simple and effective, and was implemented in real-time. Using the developed hardware with the embedded proposed algorithm, laboratory experiments and in situ field tests were performed, and results showed that the proposed approach was able to accurately determine the rock bolt length. The hardware and data processing algorithm proposed in this paper can also be adopted for property assessment of other types of embedded structures.

## Figures and Tables

**Table 1:** The results for five of the chosen rock bolts in a highway tunnel in Wuqi County.

Rock Bolt Number	Measured Length (m)	Actual Length (m)	Error (%)	Average Error (%)
1	2.01	2.00	0.5	0.8
2	3.05	3.10	1.6	
3	3.16	3.15	0.3	
4	2.82	2.80	0.7	
5	2.92	2.95	1.0	

**Table 2:** The results for five of the chosen rock bolts at Yangqu Hydropower Station.

Rock Bolt Number	Measured Length (m)	Actual Length (m)	Error (%)	Average Error (%)
1	2.02	2.00	1.0	1.0
2	1.81	1.80	0.6	
3	2.77	2.80	1.0	
4	3.76	3.80	0.7	
5	3.15	3.10	1.6	

## References

[1] Beard M., Lowe M. (2003). Non-destructive testing of rock bolts using guided ultrasonic waves. Int. J. Rock Mech. Min. Sci..

[2] Madenga V., Zou D., Zhang C. (2006). Effects of curing time and frequency on ultrasonic wave velocity in grouted rock bolts. J. Appl. Geophys..

[3] Zou D., Cui Y., Madenga V., Zhang C. (2007). Effects of frequency and grouted length on the behavior of guided ultrasonic waves in rock bolts. Int. J. Rock Mech. Min. Sci..

[4] Lee J., Kim H., Lee I., Han S., Lee Y. (2007). Rock Bolt Integrity Evaluation in Tunnelling Using Ultrasonic NDT Techniques. Underground Space–The 4th Dimension of Metropolises, Three Volume Set+ CD-ROM: Proceedings of the World Tunnel Congress 2007 and 33rd ITA/AITES Annual General Assembly, Prague, May 2007.

[5] Han S.-I., Lee I.-M., Lee Y.-J., Lee J.-S. (2009). Evaluation of rock bolt integrity using guided ultrasonic waves. Geotechn. Test. J..

[6] Zou D.S., Cheng J., Yue R., Sun X. (2010). Grout quality and its impact on guided ultrasonic waves in grouted rock bolts. J. Appl. Geophys..

[7] Zou D., Cui Y. (2011). A new approach for field instrumentation in grouted rock bolt monitoring using guided ultrasonic waves. J. Appl. Geophys..

[8] Cui Y., Zou D. (2012). Assessing the effects of insufficient rebar and missing grout in grouted rock bolts using guided ultrasonic waves. J. Appl. Geophys..

[9] Lee I.-M., Han S.-I., Kim H.-J., Yu J.-D., Min B.-K., Lee J.-S. (2012). Evaluation of rock bolt integrity using fourier and wavelet transforms. Tunn. Undergr. Space Technol..

[10] Yu J.-D., Bae M.-H., Lee I.-M., Lee J.-S. (2012). Nongrouted ratio evaluation of rock bolts by reflection of guided ultrasonic waves. J. Geotechn. Geoenviron. Eng..

[11] Wang C., He W., Ning J., Zhang C. (2009). Propagation properties of guided wave in the anchorage structure of rock bolts. J. Appl. Geophys..

[12] Bao X.K., Li Y. (2012). Application of Stress Wave Reflection Method in Nondestructive Testing of Bolt; Advanced Materials Research.

[13] Sun B., Zheng X.T., Zeng S. (2013). Signal Feature Analysis of Excited Stress Waves in Bolts Embed in Different Anchor Medium; Advanced Materials Research.

[14] Sun X.-Y., Wang Z.-Y., Kang F.-N., Li W.-F. Method of Rock Bolts Parameters Detection Based on Harmonic Wavelet Packet Transform. Proceedings of the 2015 International Conference on Machine Learning and Cybernetics (ICMLC).

[15] Starkey A., Ivanovic A., Neilson R.D., Rodger A.A. (2003). Using a lumped parameter dynamic model of a rock bolt to produce training data for a neural network for diagnosis of real data. Meccanica.

[16] Olabi A.-G., Grunwald A. (2008). Design and application of magnetostrictive materials. Mater. Des..

[17] Kwun H., Kim S.Y., Light G.M. (2011). Magnetostrictive sensor technology for long-range guided wave inspection and monitoring of pipe. NDT Techn..

[18] Tse P., Liu X., Liu Z., Wu B., He C., Wang X. (2011). An innovative design for using flexible printed coils for magnetostrictive-based longitudinal guided wave sensors in steel strand inspection. Smart Mater. Struct..

[19] Liu Z., Zhao J., Wu B., Zhang Y., He C. (2010). Configuration optimization of magnetostrictive transducers for longitudinal guided wave inspection in seven-wire steel strands. NDT E Int..

[20] Choi S., Cho H., Lissenden C.J. (2016). Selection of shear horizontal wave transducers for robotic nondestructive inspection in harsh environments. Sensors.

[21] Xu J., Sun Y., Zhou J. (2016). Research on the lift-off effect of receiving longitudinal mode guided waves in pipes based on the villari effect. Sensors.

[22] Xu J., Wu X., Kong D., Sun P. (2015). A guided wave sensor based on the inverse magnetostrictive effect for distinguishing symmetric from asymmetric features in pipes. Sensors.

[23] Xu J., Wu X., Cheng C., Ben A. (2012). A magnetic flux leakage and magnetostrictive guided wave hybrid transducer for detecting bridge cables. Sensors.

[24] Kim H.J., Lee J.S., Kim H.W., Lee H.S., Kim Y.Y. (2014). Numerical simulation of guided waves using equivalent source model of magnetostrictive patch transducers. Smart Mater. Struct..

[25] Sheykholeslami M.R., Hojjat Y., Cinquemani S., Ghodsi M., Karafi M. (2016). An approach to design and fabrication of resonant giant magnetostrictive transducer. Smart Struct. Syst..

[26] Dai X., Wen Y., Li P., Yang J., Li M. (2011). Energy harvesting from mechanical vibrations using multiple magnetostrictive/piezoelectric composite transducers. Sens. Actuators A Phys..

[27] Liangjun Z., Jingtao W., Guocheng L. (2002). Application of wavelet transform in integrity testing of piles. Chin. J. Rock Mech. Eng..

[28] Luo M., Wang J., Xu F., Zhang H. (2012). Real-time analysis system of audio-frequency stress wave. J. Vib. Meas. Diagn..

[29] Mallat S. (1999). A Wavelet Tour of Signal Processing.

[30] Selesnick I.W. (2001). Hilbert transform pairs of wavelet bases. IEEE Signal Process. Lett..

